# PD-L1 CPS in gastroesophageal cancer: differences in routine care versus Checkmate 649 and implications for biopsy-site choice and assay standardisation

**DOI:** 10.1038/s41416-026-03404-2

**Published:** 2026-04-18

**Authors:** Lucy Flanders, Thomas Savy, Miriam Ficial, Narjis Al-Ghraibawi, Louise Barber, Sarah Slater, Rille Pihlak, David Propper, Sultana Begum, Manuel Rodriguez-Justo, Marco Gerlinger

**Affiliations:** 1 Barts Cancer Institute, Centre for Tumour Biology, Queen Mary University of London, London, UK; 2 St Bartholomew’s Hospital, Gastrointestinal Cancer Centre, London, UK; 3 Barts Health NHS Trust, Department of Cellular Pathology, London, UK; 4 Barts Cancer Institute, Centre for Tumour Microenvironment, Queen Mary University of London, London, UK; 5 University College London Hospital, Department of Pathology, London, UK

**Keywords:** Gastric cancer, Oesophageal cancer, Tumour biomarkers, Cancer immunotherapy, Metastasis

## Abstract

**Purpose:**

To explore why PD-L1 scores in metastatic gastro-esophageal adenocarcinomas (GEAs) were significantly lower in a real-world cohort compared with the CheckMate 649 (CM649) trial.

**Methods:**

PD-L1 combined positive scores (CPS) were evaluated using validated assays in 100 consecutive patients with advanced/metastatic GEA at St Bartholomew’s Hospital (SBH) and compared with CM649 (*n* = 1567). Clinicopathological factors and biopsy site were analysed to assess their impact on PD-L1 results.

**Results:**

CPS ≥ 5 was substantially less frequent in SBH patients (30%) compared with CM649 (61%). Older age ( ≥ 65 years), non-diffuse histology, and MMR deficiency were associated with CPS ≥ 5 across both cohorts, yet these factors were more common at SBH and therefore did not explain the lower positivity rate. Metastatic biopsies were more frequent in CM649 (21% vs. 9%), but CPS ≥ 5 was lower in metastases (50%) than in primary tumors (60%). Importantly, PD-L1 positivity varied by metastatic site: lymph node metastases showed the highest rate (80%), while liver (50%) and other sites (44%) were significantly lower than primaries (60%).

**Conclusion:**

PD-L1 CPS is shaped by clinicopathological context and biopsy site. The persistently lower CPS ≥ 5 prevalence at SBH despite validated testing highlights assay variability and reinforces the urgent need for assay standardisation. Preferential use of primary tumor tissue may help reduce metastasis-specific bias.

## Background

The Checkmate 649 (CM649) phase 3 trial established nivolumab in combination with a fluoropyrimidine and oxaliplatin chemotherapy as a new standard of care for the first-line treatment of patients with human epidermal growth factor receptor 2 (HER2) negative advanced or metastatic gastroesophageal adenocarcinomas (GEAs) [1]. Overall survival (OS) was significantly improved with nivolumab in the intention to treat analysis (13.8 vs. 11.6 months). Biomarker analyses revealed the clearest benefit in the 61% of patients with tumors with a PD-L1 Combined Positive Score (CPS) ≥ 5, where the addition of nivolumab increased OS to 14.4 vs. 11.1 months. This trial led to licensing of nivolumab in over 50 countries, by the FDA regardless of CPS and by the EMEA for patients with PD-L1 CPS ≥ 5. Subsequently, the Keynote 859 (KN859) phase 3 trial showed that the addition of pembrolizumab to doublet chemotherapy improved OS in the ITT population (12.9 vs. 11.5 months) and in the 78% of patients with CPS ≥ 1 (13.0 vs. 11.4 months) [2]. Pembrolizumab was approved by the FDA regardless of PD-L1 CPS and by the EMEA for patients with CPS ≥ 1. PD-L1 expression analysis by immunohistochemistry (IHC) according to the CPS method [3]. is hence critical in many countries to determine patient eligibility for the addition of immunotherapy.

We present retrospectively collected audit data from 100 consecutive GEA patients at St Bartholomew’s Hospital (SBH) cancer centre which showed a significantly lower proportion of PD-L1 CPS ≥ 5 than expected based on CM649. Clinicopathological features such as MMR status [4–6] or age [7] have been demonstrated to influence PD-L1 expression in GEA and results can differ between primary tumors and metastases [8–10]. Thus, we subsequently performed a post-hoc analysis of individual patient data from > 1500 patients from the CM649 trial to assess whether differences in the population under study or the location of biopsy site influence can explain the lower PD-L1 scores. In this large and well-annotated clinical trial cohort, we define clinical and pathological characteristics that significantly associate with PD-L1 CPS ≥ 5, provide insights into the biology of PD-L1 expression, and we identified a need to improve CPS assay standardisation.

## Methods

100 consecutive patients with GEA since routine PD-L1 testing started at SBH in January 2023 until October 2024 were selected as a cohort representative of the population seen at our centre, and their PD-L1 CPS results were collected retrospectively. No formal power calculation was performed. This was done as part of a service evaluation audit of PD-L1 biomarker results at SBH. Ethics board review and approval by an external review board was therefore not required as per Health Research Authority Guidance. PD-L1 staining and scoring had been performed with a clinically validated test in a large commercial UK accredited medical laboratory. Analysis with the pharmDx 28-8 antibody assay, which was also use in the CM649 trial, was routinely requested. A smaller proportion of patients which had testing requested by referring clinicians were analysed with the pharmDx 22C3 antibody assay. CPS ≥ 5 was used as the cutoff in view of the EMEA license for nivolumab. Patient demographics including sex, age and ethnicity along with relevant clinicopathological characteristics were extracted from the SBH electronic patient records. Characteristics of SBH patients were first compared to the aggregate data from the patients in the nivolumab plus chemotherapy and chemotherapy arms from the published table in the CM649 paper [1]. To assess whether these populations differed. To gain further insight into the differences and determine whether specific demographic or clinicopathological features were associated with PD-L1 CPS ≥ 5, the CM649 individual patient clinical trial data was made available by Bristol Myers Squibb on the VIVLI platform (Vivli ID 9817), analysed within the research environment and results were downloaded for publication after approval was obtained from the trial sponsor. PD-L1 CPS data in CM649 was recorded as <1, 1, and multiples of 5 whereas reporting was with integer numbers for the SBH cohort. GraphPad Prism 10 was used to perform Chi Squared, Fishers Exact and Mann-Whitney U tests to assess statistical significance where *p*-values < 0.05 were considered statistically significant. Progression-free survival was analysed in patients who had biopsies from primary tumors vs. metastases with the Kaplan-Meier method, where an event represents disease progression or death and censor data point representing the last follow-up. RStudio v4.5.0 was used to perform the Log rank test for statistical analysis.

## Results

### PD-L1 positivity and clinical characteristics in the SBH cohort versus CM649

Data from 100 consecutive patients with advanced or metastatic GEA who had PD-L1 testing at SBH was collected. This patient cohort was first compared to published data for 1581 patients from the CM649 trial [1]. (Table 1). Seventy five percent of SBH samples had been analysed with the 28-8 pharmDx antibody which has been used as the companion diagnostic in CM649, the 22C3 pharmDx antibody was used for 25%. Only 30% of SBH patients had a PD-L1 CPS ≥ 5 compared to 61% of patients in CM649. This was statistically significant (*p* = <0.001). Thus, a substantially smaller fraction of patients with advanced or metastatic GEA was eligible for the addition of nivolumab at our centre than expected based on the CM649 trial. The proportion of tumors that was PD-L1 positive (defined as CPS ≥ 1) in the SBH population (59%) was also significantly smaller compared to CM649 (83%, *p* = <0.001). We hence compared the demographic and clinicopathological characteristics of SBH patients to those from the CM649 publication [1]. to assess whether these populations differed (Table 1).

**Table 1:** Clinicopathological characteristics of 100 patients with PD-L1 CPS results from SBH in comparison to the data of the 1581 patients in CM649 in the chemotherapy alone and chemotherapy and nivolumab arm from [1].

	Patient characteristic	Locally advanced/Metastatic disease *n* = 100	All patients (Nivolumab + chemotherapy and chemotherapy alone) *n* = 1581	
	**TOTAL**	**n (%)**	**n (%)**	**p-value**
**PD-L1 CPS scores**	<1	41 (41)	265 (17)	*p* = <0.001
	≥1	59 (59)	1297 (82)	
	<5	70 (70)	607 (38)	*p* = <0.001
	≥5	30 (30)	955 (61)	
	not evaluable	0 (0)	19 (1)	
**Antibody used**	28 8	75 (75)	1581 (100)	*p* = <0.001
	22 C3	25 (25)	0 (0)	
**Age**	<65	46 (46)	961 (61)	*p* = 0.003
	≥65	54 (54)	620 (39)	
**Sex**	male	60 (60)	1100 (70)	*p* = 0.058
	female	40 (40)	481 (30)	
**Ethnicity**	White	61 (61)	1097 (69)	*p* = <0.001
	Asian	19 (19)	375 (24)	
	other	20 (20)	109 (7)	
**Primary tumour location at diagnosis**	oesophageal	30 (30)	211 (13)	*p* = <0.001
	GOJ	16 (16)	260 (16)	
	gastric	53 (53)	1100 (70)	
**Number of organs with metastases**	1	58 (58)	344 (22)	*p* = <0.001
	≥2	42 (42)	1237 (78)	
**Site of metastases**	liver	28 (28)	615 (39)	*p* = <0.001
	peritoneum	40 (40)	377 (24)	
**Histology (Lauren classification)**	intestinal	37 (37)	539 (34)	*p* = 0.936
	mixed	7 (7)	106 (7)	
	diffuse	32 (32)	527 (33)	
	unknown	24 (24)	409 (26)	
**MSI/MMR status**	MSS/proficient	91 (91)	1377 (87)	*p* = 0.032
	MSI- H/deficient	7 (7)	44 (3)	
	unknown	2 (2)	158 (10)	

Statistical analysis performed with Chi Squared and Fishers Exact test.

Unsurprisingly for a comparison of a cohort from routine clinical care versus a clinical trial, several patient characteristics significantly differed. A larger proportion of SBH patients was over the age of 65 (*p* = 0.003) and of ethnicity other than White or Asian (*p* = < 0.001). Primary tumors in the oesophagus were more common and, in the stomach, less common (*p* < 0.001) than in CM649. Most patients at SBH had a single metastatic organ site compared to 2 or more being the commonest in CM649 (*p* < 0.001). Peritoneal disease was more common and liver metastases less common in SBH patients than in CM649 (*p* = 0.001). Although MMR/MSI status differed significantly (*p* = 0.032), the MMR-deficient (MMRd) subgroups were small in both cohorts.

### Individual patient level analysis of the SBH and CM649 cohorts

**Fig. 1:**
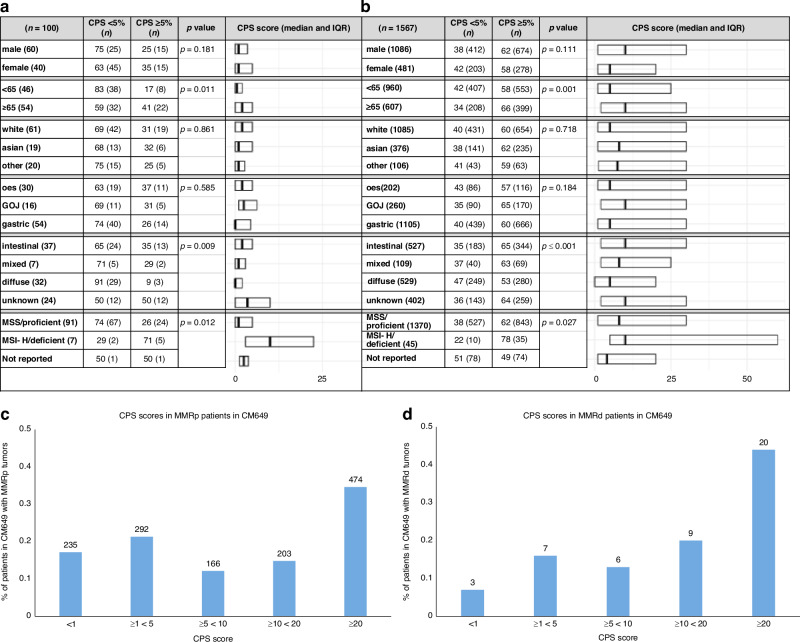
Associations of PD-L1 CPS with clinico-pathological characteristics. **a** SBH cohort and **b** CM649 patient level data. CM649 data is from the chemotherapy and chemotherapy-nivolumab arms. Boxplots show the median (bold vertical bar) and interquartile ranges (IQR) of PD-L1 CPS. Statistical analysis performed with Chi Squared Test. PD-L1 CPS in CM649 according to MMR status. **c** MMRd tumors **d** MMRp tumors.

We next sought to determine whether specific demographic or clinicopathological features were associated with PD-L1 CPS ≥ 5, and whether different prevalences of these features could account for the lower PD-L1 CPS positivity in the SBH cohort compared to CM649. Individual patient-level data from the CM649 trial was obtained for this post-hoc analysis. In the SBH cohort (Fig. 1a), the proportion of patients with CPS ≥ 5 was significantly higher in patients aged ≥ 65 years compared to those < 65 years (*p* = 0.011). CPS ≥ 5 also varied significantly by histological subtype (intestinal (35%), mixed (21%), and diffuse (9%), *p* = 0.009) and was more frequent in MMRd tumors than in MMRp tumors (71% vs. 26% respectively, *p* = 0.012). The same three characteristics were significantly associated with higher PD-L1 CPS in the CM649 cohort (Fig. 1b): Patients aged ≥65 years had a higher proportion of PD-L1 CPS ≥ 5 tumors than those < 65 (66% vs. 58%, *p* = 0.001). Histological subtype was significantly associated with PD-L1 CPS ≥ 5, following the same pattern as in the SBH cohort (intestinal (65%), mixed (63%), and diffuse (53%), *p* = < 0.001). PD-L1 CPS ≥ 5 was more frequent in MMRd tumors compared to MMRp (78% vs. 62%, *p* = 0.012), although the difference was less pronounced than in the SBH cohort. To further characterise the impact of MMRd, we evaluated a broader range of CPS cut-offs. This showed a stronger skew of MMRd (Fig. 1c) towards higher PD-L1 CPS than in the MMRp group (Fig. 1d).

Taken together, this analysis identified the same three factors; older age, MMRd tumors, and non-diffuse histological subtypes, to be consistently associated with PD-L1 CPS ≥ 5 across both cohorts. Importantly, these characteristics were either more common (MMRd, age ≥ 65 years) in the SBH cohort or had similar frequency (histological subtypes) in SBH and the CM649 trial (Table 1). Thus, these characteristics cannot explain the lower PD-L1 CPS ≥ 5 proportion at SBH compared to CM649. Therefore, we next investigated whether the use of distinct biopsy sites could explain this difference.

### PD-L1 CPS in primary tumors vs metastatic sites

Within the SBH cohort, 91% of patients had biopsies that were used for PD-L1 testing taken from the primary tumor, and 9% from metastatic sites. To be eligible for CM649, the trial protocol stipulated that the tumor tissue for PD-L1 testing must have been taken within 6 months of enrolment [1]. In view of this, we hypothesised that a greater proportion of biopsies may have been taken from metastatic sites and that this may provide the reason for the variation seen, particularly in patients who had recurrences after surgery for early-stage disease which typically occurs 6–24 after the initial diagnosis.

**Fig. 2:**
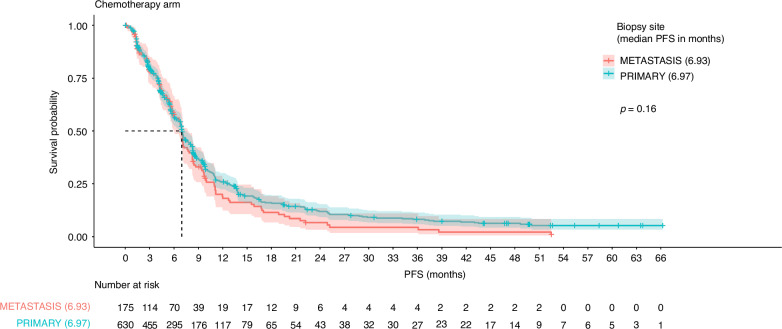
Progression free survival of patients who had PD-L1 CPS analysed biopsies from primary tumors vs. metastases. Kaplan-Meier Plot of patients on the chemotherapy arm of CM649. Dotted lines indicate the median PFS. Red and blue corridors are 95% confidence intervals. Log-rank test was used for statistical analysis.

Indeed, a significantly higher percentage of patients in CM649 had PD-L1 testing performed on a biopsy from a metastasis (21%, *n* = 325) than in the SBH cohort (*p* = 0.005). However, PD-L1 CPS ≥ 5 was significantly less common in biopsies taken from metastases than in those taken from primary tumors in CM649 (50% vs. 60%, *p* = 0.001). The more frequent use of tissue from metastases for CPS analyses does hence not account for the differences in CPS between CM649 and the SBH cohort. As these were not paired primary tumor and metastatic biopsies from the same patients, we questioned whether patients who had the primary tumor biopsied and those where a metastasis was biopsied differed with respect to clinical characteristics that may influence PD-L1 CPS (Table 2). PD-L1 testing was more frequently performed on metastatic tissue in white patients, those under 65 years of age, females, and individuals with oesophageal or gastro-oesophageal junction (GOJ) primaries (Table 2). Out of these, age < 65 years was the only characteristic associated with lower PD-L1 CPS in our analyses (Fig. 1). Additionally, progression free survival in the CM649 chemotherapy-only arm did not significantly differ for patients whose CPS was assessed from primary tumors versus metastatic sites, showing that these are prognostically similar groups (Fig. 2). Collectively, these findings support the interpretation that PD-L1 CPS ≥ 5 is consistently less frequent in metastases than in primary tumor samples, independent of other clinical or demographic variables.

**Table 2:** Clinicopathological characteristics of patients where PD-L1 CPS was analysed on primary tumor biopsies vs. metastatic sites.

	primary site biopsy *n* = 1242 (%)	metastatic site biopsy % (*n* = 325)	*p*-value
**male**	883 (71)	203 (62)	*p* = 0.003
**female**	359 (29)	122 (38)	
**< 65**	744 (60)	216 (66)	*p* = 0.031
**≥ 65**	498 (40)	109 (34)	
**white**	830 (67)	255 (78)	*p* = < 0.001
**asian**	323 (26)	53 (16)	
**other**	89 (7)	17 (5)	
**oes**	147 (12)	55 (17)	*p* = 0.038
**GOJ**	204 (16)	56 (17)	
**gastric**	891 (72)	214 (66)	
**intestinal**	424 (34)	103 (32)	*p* = 0.652
**mixed**	89 (7)	20 (6)	
**diffuse**	411 (33)	118 (36)	
**unknown**	318 (26)	84 (26)	
**MSS/proficient**	1101 (97)	269 (97)	*p* = 0.952
**MSI- H/deficient**	36 (3)	9 (3)	

Clinicopathological characteristics of patients where PD-L1 CPS was analysed on primary tumor biopsies vs metastatic site biopsies in CM649 patients in the chemotherapy and chemotherapy and nivolumab arms. Statistical analysis performed using the Chi Squared test.e biopsies in CM649 patients in the chemotherapy and chemotherapy and nivolumab arms. Statistical analysis performed using the Chi Squared test. xc.

### Analysis of specific metastatic organ sites in CM649

**Fig. 3:**
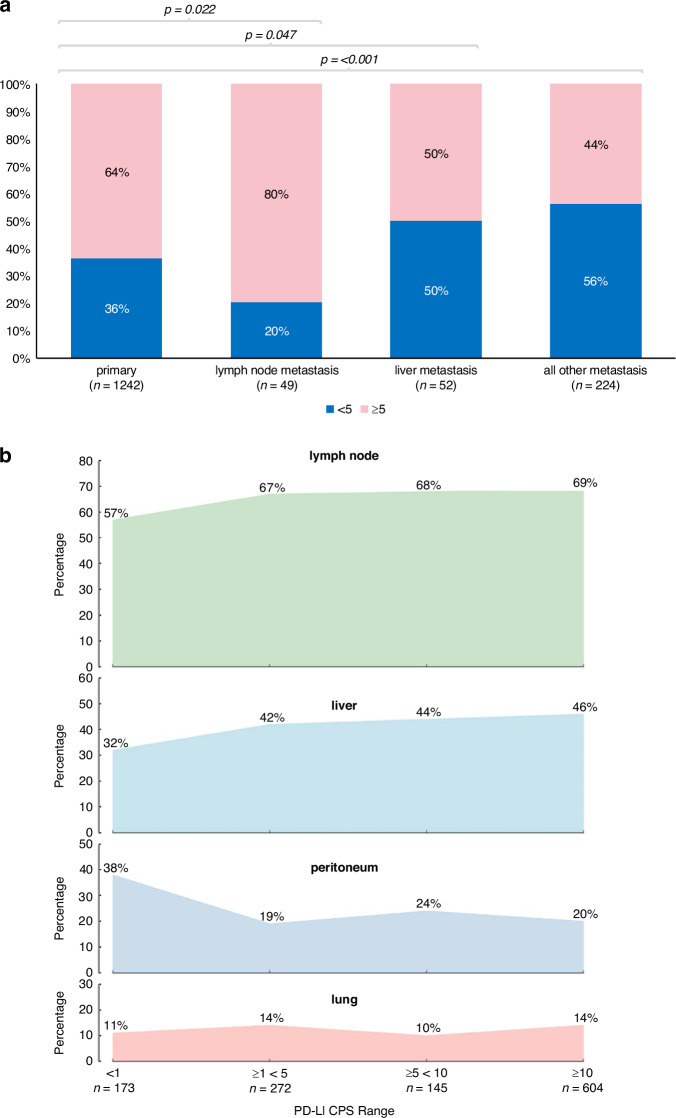
PD-L1 scores by biopsy site. **a** Proportions of patients with PD-L1 CPS ≥ 5 and CPS < 5 in patients according to biopsy site in the chemotherapy and chemotherapy nivolumab arm of CM649. **b** Prevalence of metastases in the four most common metastatic sites recorded as per baseline BICR data in CM649 in patients who had CPS analysed in the primary tumor.

We next evaluated whether PD-L1 CPS differed according to the specific metastatic organ site biopsied in patient-level data from the CM649 trial. Among the 15 reported metastatic organ sites that were biopsied in 325 patients, only liver (*n* = 52) and lymph node (*n* = 49) biopsies had sufficient sample sizes for meaningful comparison; the remaining sites were grouped as ‘other’ (*n* = 224). Lymph node biopsies demonstrated significantly higher PD-L1 positivity (CPS ≥ 5 in 78% vs. 63% in primary tumors; *p* = 0.022), whereas biopsies from the liver and from other metastatic sites showed significantly lower positivity (liver: 50% vs. 63%, *p* = 0.048; other: 44% vs. 63%, *p* < 0.001) compared to primary tumor biopsies (Fig. 3a). This suggests that biopsy site selection influences PD-L1 results, potentially affecting treatment eligibility in healthcare systems that require PD-L1 CPS ≥ 5 for nivolumab administration.

The difference in CPS score between lymph node, liver, and other metastases led us to question whether PD-L1 CPS in the primary tumor influenced which specific organs a tumor had metastasized to. The blinded independent central review (BICR) radiology data was used to determine the presence or absence of metastases across 35 anatomical sites at baseline in the CM649 patients. The most commonly involved organs, each observed in more than 5% of patients, were the lymph nodes, liver, peritoneum, and lung. The prevalence of metastases in each of these sites was correlated with PD-L1 CPS in the primary tumor (Fig. 3b). The probability of liver and lymph node metastases increased with higher CPS. The inverse relationship was observed for peritoneal metastases. No consistent association was observed between lung metastases and CPS. The largest differences in percentages of patients with a particular metastatic site were apparent between PD-L1 CPS < 1 tumors and those with PD-L1 CPS ≥ 1 < 5; a further increase in CPS did not lead to substantial changes.

Taken together, PD-L1 expression appears to influence organ-specific metastatic spread but also systematically changes in an organ-specific manner when metastases develop. For example, CPS ≥ 1 in the primary tumor associates with a higher prevalence of liver and lymph node metastases, nevertheless liver metastases tend to have lower PD-L1 expression and lymph node metastases higher PD-L1 expression than primary tumors.

## Discussion

Our audit of PD-L1 CPS in patients with advanced and metastatic GEA treated at SBH showed a substantially lower proportion of patients with CPS ≥ 5 compared to the CM649 trial (30% vs. 61%). As a result, many more SBH patients were ineligible for the addition of nivolumab to chemotherapy in the first line setting than anticipated based on trial data.

Across both cohorts, we identified age, Lauren histological subtype and MMR status as covariates significantly associated with PD-L1 CPS. Furthermore, PD-L1 CPS ≥ 5 was more frequently observed in primary tumor biopsies than in metastatic samples. Importantly, none of the covariates associated with PD-L1 CPS ≥ 5 were more prevalent in the CM649 cohort than in SBH, suggesting these factors do not explain the lower PD-L1 positivity observed in routine clinical practice at our centre and that alternative explanations need to be sought. This largely leaves pre-analytical factors and analytical factors as potential reasons for the observed discrepancy with PD-L1 testing in clinical practice being subject to several limitations that may complicate the accurate assessment of PD-L1 expression levels. There is evidence that PD-L1 antigenicity can degrade with time [11]. particularly beyond 3 years [12]. However the median age of our samples was only 4 weeks (IQR 2–11 weeks) and the CPS did not statistically differ (*p* = 0.25) between patients that had initially been diagnosed with early-stage disease and had their archival sample used (*n* = 21, median age of sample: 37 weeks, 29% CPS > 5) versus those that were diagnosed de novo with metastatic disease (*n* = 79, median age of sample: 3 weeks, 28% CPS > 5). Similarly, how slides were cut from FFPE blocks specifically for PD-L1 testing is standardised at our centre [13]. and all PD-L1 tests used validated assays performed by an accredited pathology laboratory. There is no specific reference to the age of tissue in the interpretation manual; it purely states that ‘Tissue should be stained and interpreted as close to the time of biopsy as possible [13]. Differences in antibody clones and assay platforms may influence results. A study evaluating concordance between the 3 assays demonstrated that CPS assessed with the SP263 antibody clone generally yielded the highest scores, followed by 22C3 and then 28-8 [14]. Within the SBH cohort CPS was assessed with the 28-8 assay in 75% of patients and 25% by 22C3. In the CM649 trial 28-8 was solely used. This provided no explanation for the higher scores in CM649.

Scoring algorithms used may also have an impact on results. For example, the 28-8 pharmDx interpretation manual provides three ‘possible’ scoring methodologies for calculating the PD-L1 CPS, allowing room for variation [13]. Moreover, inconsistencies in tissue adequacy, sample type, fixation protocols, tumor heterogeneity, the dynamic nature of PD-L1 expression and both interobserver and interlaboratory variability contribute to the complexity. Intratumor heterogeneity of PD-L1 expression in GEA has led to guidance suggesting a minimum of 8 biopsies to be taken at endoscopy to ensure representative tumor tissue is harvested [15, 16]. Whether this was followed could not be assessed based on our available data from SBH.

Although the above factors and differences in the clinical characteristics of cohorts limit cross-study comparability, our results demonstrate a strong need to audit and compare PD-L1 results between centres to identify whether inter-laboratory variability, differences in tissue processing or yet unidentified patient characteristics contribute to inconsistencies in CPS results. This should foster greater assay standardisation and more stringent quality control in PD-L1 testing. This is crucial to ensure that patients who have a realistic chance of benefiting from the addition of PD-1 inhibitors to chemotherapy do not miss out. This analysis in CM649, the largest cohort where clinicopathological determinants of PD-L1 CPS have been defined, can serve as a reference dataset for future comparisons. We acknowledge that our results apply mainly to the 28-8 antibody in view of this being the predominant assay used. Whether the 22C3 clone can show similar discrepancies between routine care and Keynote cohorts should be investigated. Greater understanding around the concordance between CPS and the PD-L1 tumour area positive (TAP) scores will also be vital to determine the reproducibility of this potentially simpler method [17]. However, there is some degree of inconsistency in the literature, with studies specifically comparing the 28-2 CPS and SP263 TAP assays reporting only moderate concordance [14]. or agreement limited to cases CPS ≥ 5 [18]. Furthermore, exploratory evaluation of whether such concordance patterns are maintained across histological subtypes will be important particularly as diffuse-type tumours represent a scenario of immune rich yet low cellularity disease, which could theoretically yield low TAP and high CPS.

At a more granular level, we showed an influence of biopsy site on PD-L1 CPS score. CPS ≥ 5 was more common in biopsies from lymph nodes compared to primary tumor tissue, while liver and other metastatic site biopsies yielded lower CPS values. Although the biopsy site may be dictated by disease accessibility, it suggests that selection of an organ site such as lymph nodes for biopsy increases the probability of giving immunotherapy compared to a biopsy from other metastatic sites and even the primary tumor. Higher PD-L1 CPS in lymph nodes may be the result of more efficient recognition of tumor cells within the lymph node by the immune system [4]. An alternative explanation could be the detection of PD-L1 expression in bystander immune cells in lymph node tissue, which have no relevance in cancer recognition. In the latter case, even high scores may not lead to therapeutic benefit from immunotherapy. In view of these metastasis-specific biases, the significantly higher PD-L1 CPS across all primary tumors than metastases, and the fact that 79% of patients in CM649 had PD-L1 CPS analysed on primary tumors, which likely drove the selection of the biomarker cut-off of CPS ≥ 5 we would recommend preferential use of primary tumor tissue for PD-L1 CPS analyses. This does not imply different efficacy of immunotherapy on distinct metastatic sites.

Our analysis also sheds light on the biology of PD-L1 expression in this large multi-centre dataset of over 1,500 patients. PD-L1 CPS increased with patient age which likely indicates higher average immunogenicity in older patients. The increase in the tumor mutation burden [19, 20] and hence of neoantigens [21, 22]. With increasing age at diagnosis may contribute to this. The lower PD-L1 CPS in diffuse-type tumors in CM649 is noteworthy. This GEA subtype is also associated with poorer prognosis and chemotherapy responsiveness [23–25]. The finding of low PD-L1 CPS reinforces the need to develop alternative therapeutic strategies for this subgroup. Promising avenues include the evaluation of targets such as Claudin 18.2, which shows higher expression in diffuse type tumors [26]. Finally, even MMRd tumors showed a broad range of PD-L1 scores, with 10/45 (22%) having a CPS < 5. Studies into subtype-specific PD-L1 CPS cutoffs should hence be performed. Several of these associations have previously been suggested in smaller, single-country, and retrospective cohorts, with several using PD-L1 scoring systems that are not validated for clinical use. Epstein-Barr virus (EBV) positive GEAs have also been linked to high PD-L1 expression [5]. This data was not available from CM649 or SBH, precluding analysis.

Our findings demonstrate metastatic-specific PD-L1 CPS variation, with higher PD-L1 expression favouring the development of liver and lymph node metastases. This is consistent with reports from smaller cohorts of PD-L1 CPS heterogeneity and intrapatient discordance across primary tumors, nodal [9] and distant metastases in GEA [8, 10]. with peritoneal metastases often exhibiting low PD-L1 CPS [27]. Peritoneal metastases are thought to often occur by direct invasion into the peritoneal cavity rather than hematogenous or lymphatic spread. Peritoneal seeding may hence be less dependent on PD-L1-mediated immune evasion. Intriguingly, the declining frequency of peritoneal metastases with increasing CPS raises the possibility that high PD-L1 expression may suppress peritoneal dissemination.

While acknowledging the limitations of comparing and interpreting findings across a real-world patient cohort and a prospective randomised global trial data, which differ in their baseline characteristics, the striking disparity in CPS distribution compared to CM649 has clear implications for future research. It demonstrates the urgent need for standardisation of PD-L1 testing to ensure alignment between clinical trial data and real-world practice. Further prospective studies are warranted to clarify determinants of PD-L1 CPS expression. These insights may help optimise PD-L1 assessment and increase the likelihood of appropriately identifying patients who could benefit from immunotherapy.

### Statement of significance/Translational relevance

In many health care systems, the PD-L1 combined positive score (CPS) is a critical biomarker for the qualification of patients with advanced/metastatic gastroesophageal adenocarcinoma (GEA) for addition of immunotherapy. In this study, CPS ≥ 5 was observed in only 30% of 100 patients in routine care, markedly lower than the Checkmate 649 trial (61%). Post-hoc analysis of trial data identified older age, non-diffuse subtype, MMR deficiency, and biopsies taken from primary tumor to be associated with CPS ≥ 5. These characteristics were overrepresented in real world patients, highlighting a significant discrepancy not explained by biological/clinical cohort differences. Beyond demonstrating an impact of biopsy site selection on PD-L1 CPS results and potential need for a histological subtype-specific cut-offs, our data shows that research into pre-analytical and analytical factors impacting PD-L1 assay performance and greater assay standardisation is needed. This is crucial to ensure patients do not miss out on effective immunotherapies.

## Data Availability

The data analyzed in this study from CM649 are available with permission from Bristol Myers Squibb on the VIVLI platform. Restrictions apply to the availability of these data, which were used under license for this study. The individual patient data analysed from SBH is not publicly available due to patient confidentially.
